# *In Vivo* Dynamics of the Musculoskeletal System Cannot Be Adequately Described Using a Stiffness-Damping-Inertia Model

**DOI:** 10.1371/journal.pone.0019568

**Published:** 2011-05-27

**Authors:** Dinant A. Kistemaker, Leonard A. Rozendaal

**Affiliations:** 1 Department of Psychology, University of Western Ontario, London, Canada; 2 Research Institute MOVE, Faculty of Human Movement Sciences, VU University Amsterdam, Amsterdam, The Netherlands; Massey University, New Zealand

## Abstract

**Background:**

Visco-elastic properties of the (neuro-)musculoskeletal system play a fundamental role in the control of posture and movement. Often, these properties are described and identified using stiffness-damping-inertia (KBI) models. In such an approach, perturbations are applied to the (neuro-)musculoskeletal system and subsequently KBI-model parameters are optimized to obtain a best fit between simulated and experimentally observed responses. Problems with this approach may arise because a KBI-model neglects critical aspects of the real musculoskeletal system.

**Methodology/Principal Findings:**

The purpose of this study was to analyze the relation between the musculoskeletal properties and the stiffness and damping estimated using a KBI-model, to analyze how this relation is affected by the nature of the perturbation and to assess the sensitivity of the estimated stiffness and damping to measurement errors. Our analyses show that the estimated stiffness and damping using KBI-models do not resemble any of the dynamical parameters of the underlying system, not even when the responses are very accurately fitted by the KBI-model. Furthermore, the stiffness and damping depend non-linearly on all the dynamical parameters of the underlying system, influenced by the nature of the perturbation and the time interval over which the KBI-model is optimized. Moreover, our analyses predict a very high sensitivity of estimated parameters to measurement errors.

**Conclusions/Significance:**

The results of this study suggest that the usage of stiffness-damping-inertia models to investigate the dynamical properties of the musculoskeletal system under control by the CNS should be reconsidered.

## Introduction

It is widely acknowledged that visco-elastic properties of the (neuro-)musculoskeletal system play a fundamental role in the control of posture and movement. Visco-elastic properties arise from reflexive pathways and intrinsic properties of the muscle-tendon complex. Reflexive pathways influence dynamics through feedback from, for example, muscle spindles and Golgi-tendon organs, the gain of which is regulated by the central nervous system (CNS) [1]-[3]. Intrinsic visco-elasticity originates from the force-length-velocity relationship of the contractile element (CE) and the force-length relationship of the tendon. It has been shown that by co-contracting muscles, the CNS can adapt the properties of the musculoskeletal system to task requirements [4]–[6].

Undoubtedly, it is important to adequately describe and identify the dynamic properties of the musculoskeletal system. Often, this is done by using spring-damper-inertia (KBI) models; for example that of the ankle joint (e.g. [7]–[9]) elbow joint (e.g.[10]–[12]) or the whole arm (e.g.[13]–[15]). A KBI-model consists of a stiffness element (*K*), a damping (*B*) element, and an inertia (*I*) element. Stiffness and damping are typically identified experimentally by perturbing the human musculoskeletal system and optimizing parameter values of the KBI-model to obtain a best fit between model responses and responses observed experimentally (e.g.[9]–[14]). Using such an approach (from now on referred to as “KBI-approach”), researchers investigated which properties of the (neuro-)musculoskeletal system are controlled by the CNS.

**Figure 1:**
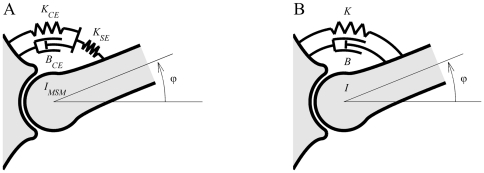
Schematic representation of the MSM model consisting of a 1 DOF segment that is actuated by a linearized Hill-type muscle model (A) and the KBI-model consisting of a 1 DOF segment actuated by a spring and damper in parallel (B).

Within the musculoskeletal system, the skeleton interacts with an elastic tendon that is in turn connected in series with a visco-elastic CE. In a KBI-model, such an elastic tendon is not present (see Figure 1). Because it is known that a tendon greatly influences the dynamics of a muscle and its interaction with the skeleton, this difference raises a critical question: what information about the musculoskeletal system is captured by the estimated stiffness and damping using a KBI-model? Unfortunately, there is no simple answer. For example, when muscle is quickly shortened, the force-velocity relationship prevents rapid length changes of the CE; thus muscle-tendon complex length change is attributed primarily to changes in tendon length. Hence the stiffness estimated will resemble tendon stiffness. For slow stretches, however, the force-velocity relationship plays a minor role and hence the identified stiffness will resemble the reciprocal of the sum of tendon and CE stiffness (CE and tendon now simply work as two springs in series). Even though KBI-models are widely used, to our knowledge, no study directly addressed which musculoskeletal properties are captured by the parameter values of KBI-models determined using the fitting-approach described above.

The purpose of the present study was threefold: i) analyzing the relation between the musculoskeletal properties and the estimated stiffness and damping, ii) analyzing how this relation is affected by the nature of the perturbation and iii) assessing the sensitivity of the estimated stiffness and damping to measurement errors. Since the real dynamical properties are not known exactly and are difficult to control in an experiment, a musculoskeletal model (MSM) was used to assess the validity of using a KBI- approach to estimate stiffness and damping *in vivo*. Our analyses show that the estimated stiffness and damping will not resemble any of the dynamical parameters of the underlying system, not even when the responses are adequately captured by the KBI-model. The analyses furthermore show that the estimated stiffness and damping will be a non-linear mixture of all the dynamical parameters of the MSM, influenced by the nature of the perturbation and the time window over which the parameters are estimated. Finally, our analyses predict a very high sensitivity of estimated parameters to measurement errors. These results suggest that the usage of stiffness-damping-inertia models to experimentally investigate the dynamical properties of the musculoskeletal system that are under the control of the CNS should be reconsidered.

## Results

### Overview

Since the real dynamical properties are not known exactly and are difficult to control in an experiment, a musculoskeletal model (MSM) was used to assess the validity of using a KBI- approach to estimate stiffness and damping *in vivo*. As mentioned in the Introduction, in the musculoskeletal system, the skeleton interacts with the contractile element through a tendon, causing the system as a whole to be of at least order three. The simplest model of such a mechanical system consists of a single-joint segment, actuated by a linearized Hill-type muscle model composed of a visco-elastic CE and a series elastic element (SE; see Figure 1A). Note that we do not want to imply that the real musculoskeletal system can be adequately represented by such a model. Actually, we think that also such a model grossly oversimplifies the musculoskeletal system. However, our rationale here is that if a KBI-model cannot describe/estimate the dynamical behaviour of a linearized Hill-type muscle model that takes into account the interaction of a compliant tendon, it can surely not do so for the real musculoskeletal system.

In essence, we used the KBI-approach to try and estimate stiffness and damping of a MSM for which the dynamical parameters are known a-priori. To do so, we followed the approach identical to those described in the literature: we perturb the system (our MSM model) and subsequently optimize the parameters of the KBI-model to obtain a best fit between the responses of the KBI-model and the MSM. This was repeated for a realistic range of values of the dynamical parameters of the MSM, for different response times and perturbations (see Materials and Methods). In addition, we also derived analytical approximations to the optimal fit between the responses of the MSM and KBI-model. This allowed us to investigate the relation between estimated and actual dynamical parameters. Finally, an analysis was carried out to assess how sensitive estimated stiffness and damping are for measurement errors.

### Responses of the MSM and the KBI-model

The dynamics of the musculoskeletal model (MSM, Figure 1a) and the KBI-model (Figure 1b) are formulated in terms of their impedance, Z*_MSM_* and Z*_KBI_* respectively, in the Laplace domain (see Materials and Methods and Supporting Information S1):
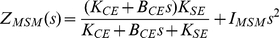
(1)

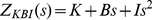
(2)


For convenience, we take the parameters of each of the models in a parameter vector and define ***p_MSM_*** = [*K_CE_ B_CE_ K_SE_ I_MSM_*]^T^ (thus: contractile element stiffness; contractile element damping; tendon stiffness; inertia) for the MSM, and ***p_KBI_*** = [*K B I*]^T^ (stiffness; damping; inertia) for the KBI-model. ***p_KBI_*** was optimized by minimizing the following error criterion function that is only sensitive to difference in the shape of the MSM (*ϕ_MSM_*) and KBI-model response (*ϕ_KBI_*):
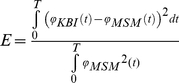
(3)


The value of *E* obtained with ***p_KBI_*** ^*^ will be indicated as E^*^ and corresponds to the (minimal) value of the error criterion function for a given set of ***p_MSM_***. *T* is the time window over which the response was fitted.

**Figure 2:**
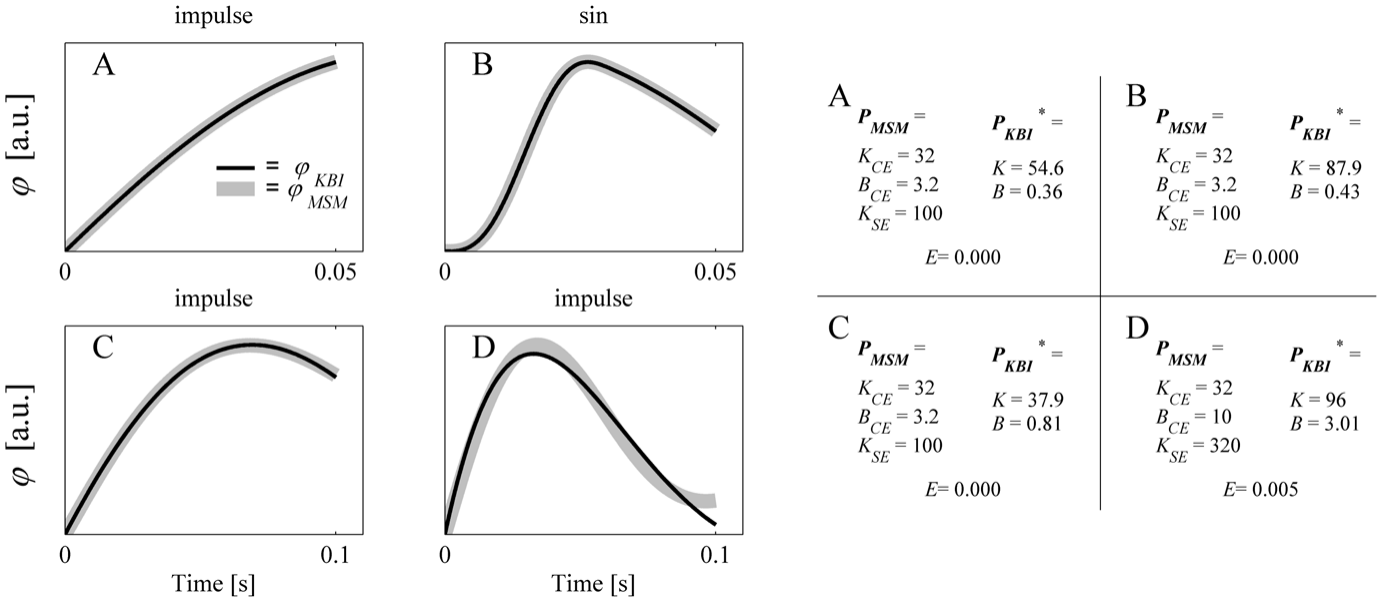
Four examples of responses of the MSM (*ϕ_MSM_*) and optimized responses of the KBI-model (*ϕ_KBI_*). The table on the right-hand side depicts the MSM parameters used and the optimized stiffness *K* and damping *B* of the KBI-model. A. Impulse responses of the MSM and optimized KBI-model. B. Same as in A, but now a sinusoid torque wave was used as a perturbation, instead of an impulse. C. Impulse response same as in A, but now the time interval over which the parameters were optimized was set to 100 ms instead of 50 ms. D. Typical example of an impulse response of the MSM for which the optimized KBI response showed marked differences.

Figure 2 shows some typical examples of responses of the MSM and that of the numerically optimized KBI-model. Figure 2A depicts the response of the MSM with parameters ***p_MSM_***  = [32 3.2 100 .1] to a torque impulse perturbation for a time window *T* of 50 ms. As can be appreciated from the figure, for this ***p_MSM_*** and response time, the KBI-model was very well capable of describing the dynamic behavior, leading to an error *E* of practically zero. The estimated stiffness *K* (55 Nm/rad) was somewhere between the CE (32 Nm/rad) and SE (100 Nm/rad) stiffness and the estimated damping *B* (0.36 Nms/rad) was about ten times lower than the CE damping (3.2 Nms/rad). Figure 2B shows the results for the exact same parameters of the MSM used in 2A, but now a high-frequency sinusoid torque wave (period 30 ms, amplitude 20 Nm; identical to that used in an experimental study of Popescu et al. [12] was used as a perturbation. Again, the KBI-model was capable of adequately describing the dynamical behavior. However, the optimized stiffness value found were markedly different (Figure 2B): 55 Nm/rad in case of an impulse perturbation and 88 Nm/rad in case of a sinusoid perturbation. Figure 2C shows the response of the MSM with the same parameters and perturbation as in Figure 2A, but now the time interval over which the response was fitted was 100 ms instead of 50 ms. Again, the KBI-model was capable of adequately describing the dynamic behavior, albeit with substantially different estimated stiffness and damping. Figure 2D shows an example of a response of the MSM (torque impulse and ***p_MSM_*** set to [32 10 320 .1]) that could not be captured well by the KBI-model (*E* = 0.005).

**Figure 3:**
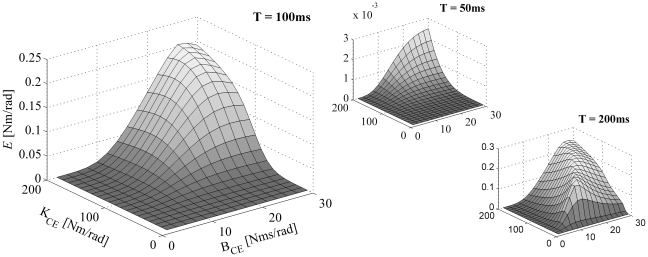
The minimal error (E^*^) between the responses of the optimized KBI-model and MSM as a function of *K_CE_* and *B_CE_*. *K_SE_* was set to 5×*K_CE_*. Results are shown for impulse torque perturbations for three different time intervals (T = 50, 100 and 200 ms) over which the responses were optimized. Every grid point in the graph represents an optimization; a total of 300: *K_CE_* ranging from 10 to 200 Nm/rad in 20 steps and *B_CE_* ranging from 2 to 30 Nms/rad in 15 steps.

Figure 3 shows a more general overview of the capability of the KBI-model to describe the dynamical behavior of the MSM for a realistic range of parameters. In this figure the matching criterion *E*, which, as stated before, captures the difference between the response of the MSM and that of the optimized KBI-model (see Eq. 3), was plotted as a function *K_CE_* and *B_CE_*. For graphical purposes, and based on the rationale that in the real musculoskeletal system *K_CE_* and *K_SE_* cannot be chosen independently (SE is a passive non-linear spring and hence its stiffness depends on the CE force), *K_SE_* was set to a fixed value of *K_CE_* (see also Methods and Materials). Figure 3 shows the results for *K_SE_* set to 5×*K_CE_*; a more general overview of the results is given in Supporting Information S1. Figure 3 shows that for some range of MSM parameters the KBI-model is less closely matching the dynamical behavior of the MSM. Nevertheless, matching errors were in general small especially for smaller time windows (see also Supporting Information S1), which is in accordance with experimental results indicating that for small operating ranges KBI-models can approximate the kinematic responses of the musculoskeletal system quite well [16]–[18]. However, as will be shown later in the sensitivity analysis, matching errors as small as 0.001 can result from a large range in values of estimated *K* and *B*.

**Figure 4:**
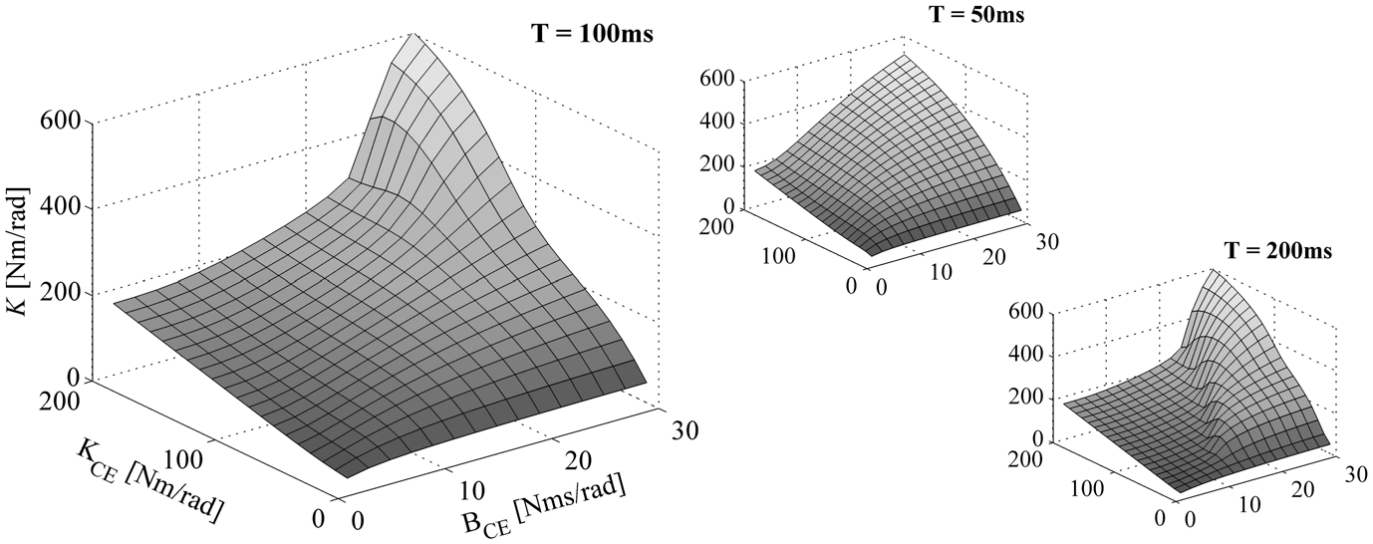
The estimated stiffness (*K*) as a function of *K_CE_* and *B_CE_*. As with Figure 3, *K_SE_* was set to 5×*K_CE_*. Results are shown for impulse torque perturbations, for three different time intervals (T = 50, 100 and 200 ms) over which the responses were optimized.

Figures 4 depicts the estimated stiffness *K* as a function of the MSM parameters. Like in Figure 3, *K_SE­_* was set to 5×*K_CE_* (see Supporting Information S1 for *K_SE­_* = 2×*K_CE_* and 10×*K_CE_*). Clearly, the estimated *K* does not have a simple relation with the dynamic parameters of the MSM, not even in the cases in which the responses of the MSM and KBI-model were virtually identical (i.e. small values of *E* in Figure 3). Only if *B_CE_* was set to zero, *K* was linearly related to CE and SE stiffness (the stiffness of CE and SE then work like two springs in series). For all other (more realistic) combinations of MSM parameters, estimated stiffness for a fixed perturbation depended non-linearly on all parameters of the MSM. For example, for a fixed *K_CE_* of the MSM, estimated stiffness could range from 1 to 6 times *K_CE_*, depending on the *damping* of the CE.

**Figure 5:**
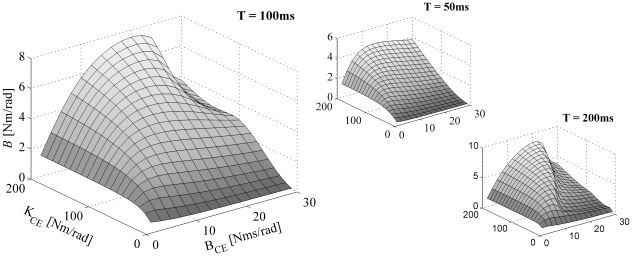
Same as **Figure 4**, but now estimated damping (*B*) is shown as a function of *K_CE_* and *B_CE_*.

Figure 5 shows the estimated damping *B* as a function of the MSM parameters. The resemblance between CE damping and estimated damping is in general very non-linear. More importantly, the damping estimated depends greatly on the stiffness of the CE and SE. By changing CE *stiffness*, but not its damping, estimated damping can change 100-fold! Furthermore, the estimated stiffness and damping in general do not resemble any of the parameters of the musculoskeletal model, not even in those cases for which the responses of the KBI-model were virtually identical to those of the MSM. Above that, estimated stiffness and damping greatly depended on the time interval over which the response were optimized and on the type of perturbation applied.

### Analytically approximated KBI parameters

As can be appreciated from Figures 2, 3, 4, 5 and Supporting Information S1, estimated parameters depend non-linearly on all parameters of the MSM. To gain insight in this relationship, we approximated the relationship between ***p_MSM_*** and ***p_KBI_*** analytically for low and high frequencies (see Methods and Materials). Table 1 gives the stiffness and damping for the low frequency approximation ([*K_LF_ B_LF_*]) and high frequency approximation ([*K_HF_ B_HF_*]).

**Table 1:** Analytical low and high frequency approximations of KBI parameters.

*Low frequency*	*High frequency*
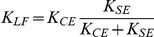	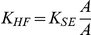
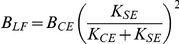	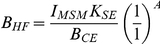

Under low-frequency conditions, the identified stiffness *K_LF_* is obtained by the well-known expression for the stiffness of two springs connected in series. As tendon stiffness in reality never reaches infinity, the identified stiffness is always lower than the CE stiffness. (If *K_CE_* is negative, as might occur in muscles above optimum length, and assuming that ||*K_SE_*||>||*K_CE_*||, CE yielding tends to be amplified by the SE.) In the expression for the estimated damping *B_LF_*, the same factor *K_SE_* /( *K_CE_*+*K_SE_*) occurs to the power of two. This means that SE stiffness reduces the estimated damping even more than the estimated stiffness.

Under high-frequency (HF) conditions, we found a very different set of parameters for the KBI-model. The effective stiffness corresponds to the SE stiffness. This can be readily understood as for very fast perturbations CE damping prevents elongation of the CE. In that case, all muscle length changes is due to changes in tendon length and hence stiffness estimated is the stiffness of the tendon. The expression for the estimated damping constant *B_HF_* is quite remarkable: the effective damping constant *increases* with SE stiffness and *decreases* with CE damping! The higher CE damping, the less it will change its length for a given perturbation force. As a result, relatively more of the rate of change in muscle length is taken up by SE (which has zero damping), and hence estimated damping will be less. Conversely, a high SE stiffness enforces CE stretching and hence will increase the estimated damping. In general, *in vivo* estimated stiffness and damping will be somewhere between the low and high frequency approximation depending on the parameters values of the musculoskeletal system and on the type of perturbation applied.

### Sensitivity

**Figure 6:**
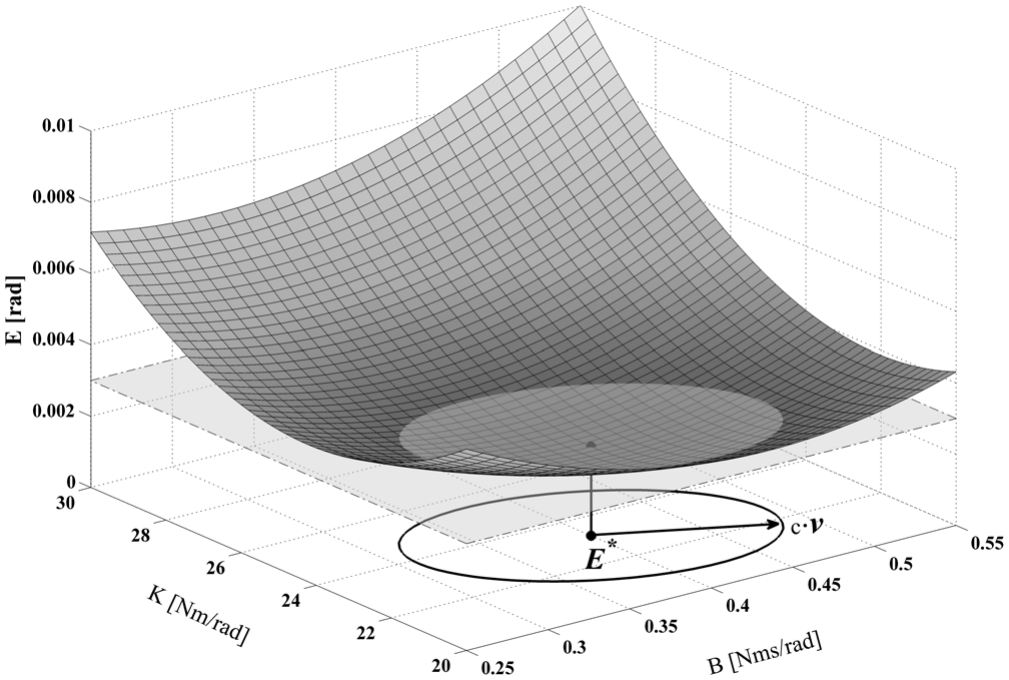
Graphical representation of the error function *E*, around its minimum E^*^ ( = 0.002 rad), as a function of *K* and *B*. In this sensitivity analysis, we aim to find, the *maximal* change in parameter values of the KBI-model that leads to a chosen increase in the optimal matching criterion E^*^. Note that this figure represents one optimization for one given set of MSM parameter values. E^*^ was estimated using numerical optimization. Every point on the surface was calculated by simulating the perturbation response of the KBI-model with the corresponding grid values of [*K B*]. Then, the value for the matching criterion *E* was calculated using that KBI response and the response of the MSM model (see Eq. 3). Also depicted in the figure is a horizontal plane corresponding to an *E* of 0.003 (thus *E* ^*^ plus a Δ*E* of 0.001), its intersection with the surface of *E* and the ellipsoid contour of this intersection in the ground plane. The direction in which the vector [*K B*] can be changed the most before *E* equals E^*^+Δ*E* = 0.003 is given by the eigenvector ***v*** belonging to the smallest eigenvalue of ***H***, i.e. the second derivative matrix of *E.* Note again that this analysis was done for every ***p_MSM_***.

In an experiment, inherent errors occur in both the applied perturbation and the measured response. To directly explore the sensitivity of estimated stiffness and damping to such errors, a sensitivity analysis was carried out. Any given difference between actual and measured joint angle time histories can be expressed by a corresponding value of the error criterion *E*. In this sensitivity analysis, we calculated the maximal change in parameter values of the KBI-model that led to an arbitrarily small Δ*E* of 0.001, and hence assessed the sensitivity of parameters that can be expected on the basis of measurement errors. In figure 6 it is explained how the maximal change in KBI parameters was found.

**Figure 7:**
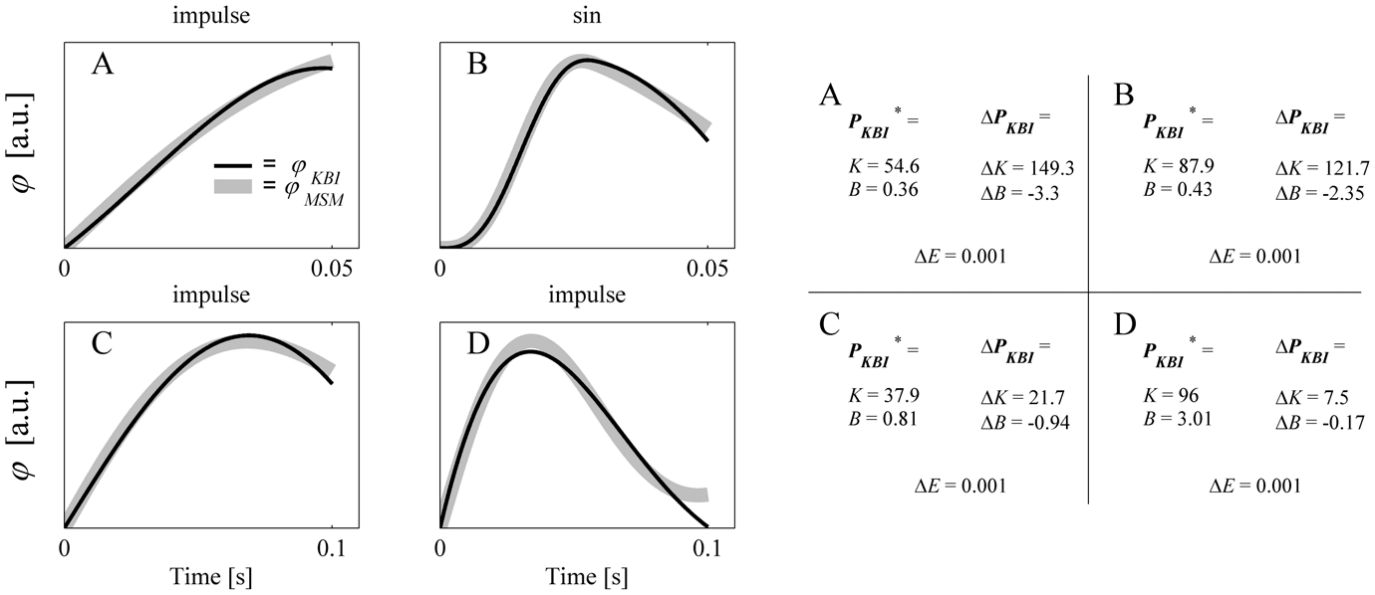
Four examples of responses of the MSM and responses of the KBI-model with a maximal change in optimal *K* and *B* leading to an increase in matching error Δ*E* = 0.001. The table gives the optimal values of *K* and *B* as well as the maximal change Δ*K* and Δ*B*. Parameters of the MSM, perturbation type and time interval over which the perturbation was optimized were identical to those of Figure 2.

Figures 7A–D show the responses of the MSM that are identical to those plotted in figure 2A–D. In contrast to figure 2, the KBI-model responses now were obtained using the optimally estimated ***p*** _KBI_^*^ plus the Δ***p*** *_KBI_* leading to a small increase in matching error Δ*E* of 0.001. Evidently, the small additional matching error of 0.001 changed almost unnoticeably the responses of the KBI-model. In an experimental study, these responses would clearly have been marked as being excellent fits of those observed experimentally. Yet, such a small difference in response can correspond to very large differences in estimated stiffness and damping. As an example (see Figure 7A), an increase in matching error of 0.001 can lead to a change in stiffness of about 200% and a change in damping of over 900%. In contrast with the value for the estimated stiffness and damping, it was found that the sensitivity to measurement errors did not depend on the type of perturbation applied.

**Figure 8:**
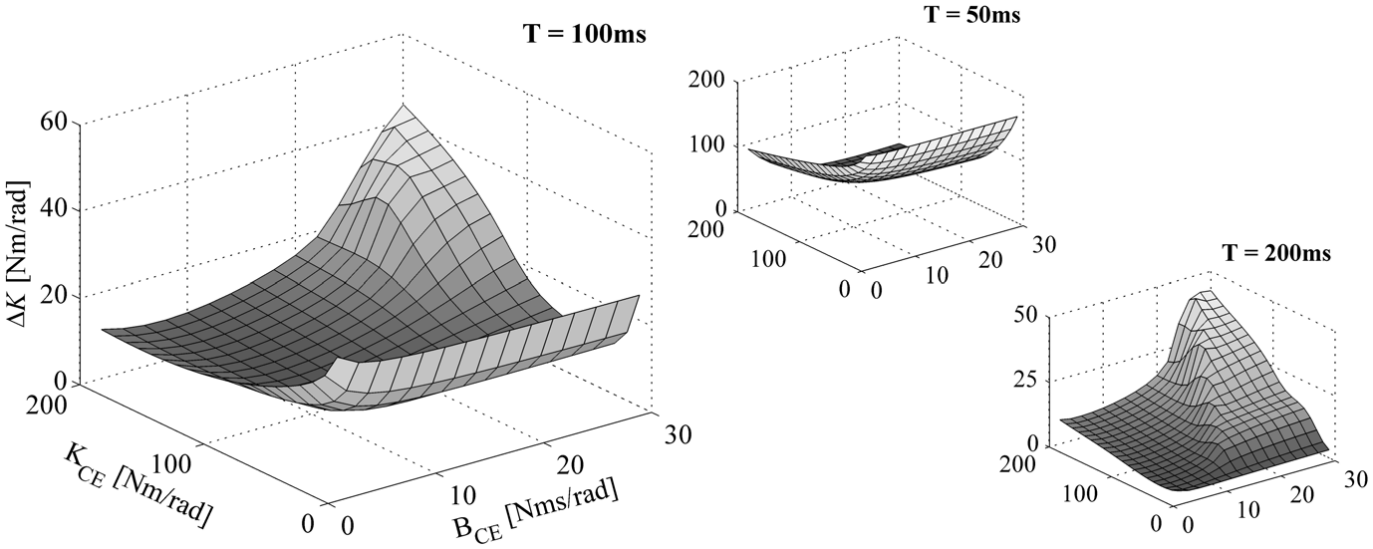
Sensitivity of estimated stiffness for measurement/optimization errors. In this figure, the maximal difference in stiffness (Δ*K*) for a small given change in matching criterion (Δ*E* = 0.001) was plotted as a function of *K_CE_* and *B_CE_*. *K_SE_* was set to 5×*K_CE_*. Results are shown for impulse torque perturbations with three different time intervals (T = 50, 100 and 200 ms) over which the responses were optimized (note the difference is scaling of the axis).

Figure 8 shows the sensitivity of the estimated stiffness for a small change in matching error (Δ*E* = 0.001) as a function of *K_CE_* and *B_CE_* (again, *K_SE_* = 5×*K_CE_*). As with the four examples depicted in Figure 7, the general sensitivity of the estimated stiffness was considerable and tended to increase with decreasing time intervals over which the responses were optimized. The mean relative change of *K* (Δ*K*/*K*) was 65% (±13%) when the time interval was set to 50 ms, and 12% (±7%) and 7% (±1%) when the time interval was set to 100 and 200 ms, respectively.

**Figure 9:**
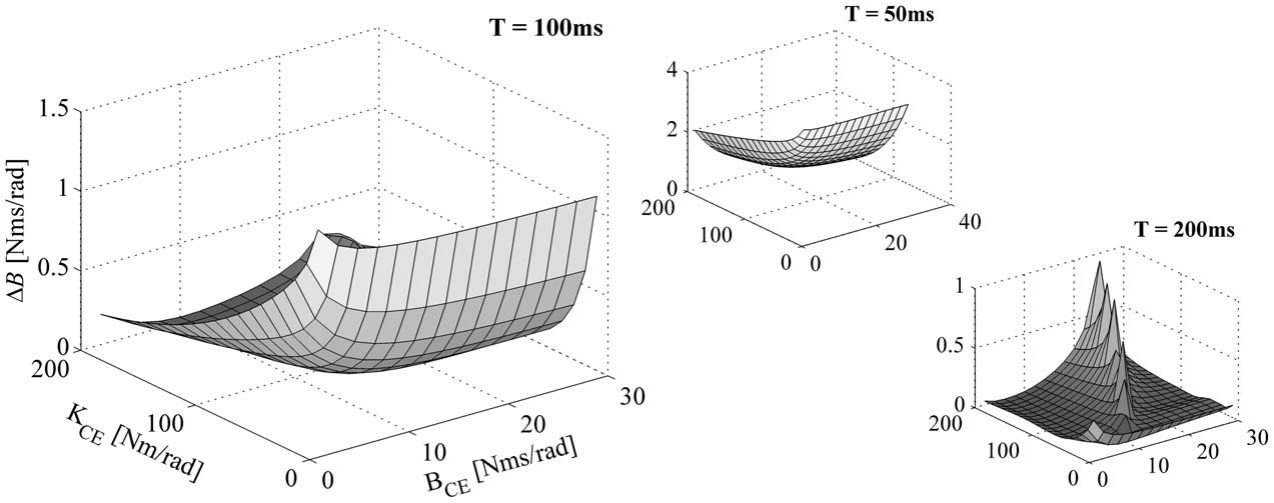
Sensitivity of estimated damping for measurement/optimization errors. Maximal difference in estimated damping (Δ*B*) is shown as a function of *K_CE_* and *B_CE_* (Δ*E* = 0.001 and *K_SE_* = 5×*K_CE_*). Results are shown for impulse torque perturbations with three different time intervals (T = 50, 100 and 200 ms) over which the responses were optimized.

Figure 9 shows the sensitivity of the estimated damping for a small change in matching error (Δ*E* = 0.001) as a function of *K_CE_* and *B_CE_* (again, *K_SE_* = 5×*K_CE_*). As with the sensitivity of the estimated stiffness, the general sensitivity of the estimated damping was considerable and tended to increase with decreasing time intervals over which the responses were optimized. The mean relative change of *B* (Δ*B*/*B*) was 880% (±1350%) when the time interval was set to 50 ms, and 65% (±99%) and 4% (±2%) when the time interval was set to 100 and 200 ms, respectively.

### Different types of perturbations

Most of the results shown were obtained using torque impulse perturbations (except for sinusoidal perturbations in Figs. 2B and 7B). Simulations were repeated for torque pulses, for torque step perturbations and for sinusoidal torque perturbations. It was found that the estimated stiffness and damping depended critically on the type of perturbation used (compare, for example, Fig. 2A and B, or 7A and B). Importantly, none of the perturbation types was superior to the others with respect to i) the ability of the KBI-model to describe the dynamical behaviour of the MSM, ii) the complexity of the relationship between the estimated parameters of the KBI-model and the parameters of the MSM and iii) the sensitivity of the estimated stiffness and damping to measurement errors.

## Discussion

The results of this study showed that the estimated stiffness and damping estimated using a KBI- model did not resemble any of the parameters of the MSM, not even when there was virtually no difference in the perturbation responses of the MSM and fitted KBI-model. In fact, the simulations and analytical analyses showed that the optimally estimated stiffness and damping depended non-linearly on all dynamical parameters of the MSM, by the type of the perturbation applied and the time interval over which the parameters were estimated. Perhaps even more problematically, the estimation of stiffness and damping was found to be extremely sensitive to measurement errors that are inherent to experiments. In other words, even very small differences in measured and fitted responses can give rise to large differences in estimated stiffness and damping. As can be appreciated from Figure 5, fits that would all be marked as excellent in an experimental study can result from changes in estimated stiffness up to 200%! The sensitivity greatly increased with smaller time intervals over which stiffness and damping were estimated (see Figure 7A and B). The reason for this is that inertia is the dominating dynamical parameter for the first part of the response. Reversely, stiffness and damping play little role in the first part of the response and hence are very sensitive to measurement errors. For time intervals as large as 200 ms, the sensitivity for measurement errors was smaller, but then the capability of the KBI-model to adequately describe the behavior of the MSM was greatly diminished.

It goes without saying that the musculoskeletal model used in this study is a gross simplification of the real musculoskeletal system. The Hill-type muscle model used in this study neglected the non-linear force-length-velocity relationship of real muscles. Above that, several other dynamical properties originating from, for example, activation dynamics [19], [20], short range stiffness [21] and history dependence of muscle force production [22] were not taken into account at all. We again want to stress that by using a linearized Hill-type muscle model we do not imply that it is capable of adequately describing the dynamics of the real musculoskeletal system. We chose such a model as it is the simplest model that takes into account the essential interaction between the stiffness of the tendon that is in series with the stiffness and damping of the contractile element that gives rise to so-called “contraction dynamics”. Our rationale here was that if the KBI-approach fails to adequately characterize and estimate the dynamical parameters of a linearized Hill-type musculoskeletal model, it must be all the more problematic for characterizing the real musculoskeletal system.

The model used in this study did furthermore not include the reflexive control loops that are known to affect the dynamics of the system. It has been suggested that feedback from muscle spindles and Golgi tendon organs might improve the linearity of the responses of the *neuro-musculoskeletal* system to perturbations (e.g. [23], [24]). However, it is debatable how much the kinematic response of the neuro-musculoskeletal to perturbations resembles that of a KBI-model. For example, in a study by Tsuji et al [25], the maximal correlation coefficient between observed responses (time window >350 ms) and that of a KBI-model was around 0.85. More importantly, as stated above and exemplified by Figure 2A–C, we have shown that the actual flaw of a KBI approach lies not in its predictability of kinematic responses to perturbations, but in the complex non-linear relationship of the estimated parameters with those of the underlying system, and the very high sensitivity of the estimated parameters to measurement errors. Taken together, the results of the present study indicate that attempting to capture the dynamics of the neuro-musculoskeletal system using a second order stiffness-damping-inertia model is both theoretically and empirically problematic. Yet, this method of estimating stiffness and damping is widely used.

Our finding that the estimated dynamical parameters depend greatly on i) the nature of the perturbation, ii) the time interval over which the response is being fitted and iii) are very sensitive to measurement errors is supported by the very wide range of joint stiffness reported in the literature. For example, elbow joint stiffness values reaches from about 14 Nmrad-1 to 126 Nmrad-1 in ‘do resist’ paradigms (e.g. [11], [13], [26]). Based on the rationale described above, this study predicts a very high variability of the stiffness and damping estimated in such studies. Unfortunately, most of the studies that reported in vivo stiffness and damping estimates do not present relevant measures of variability. In one exception, Popescu et al. [12] assessed the range of possible *K* and *B* values which yielded fits (to 30 ms sinusoid torque perturbations over a time interval of 50 ms) within the standard error bounds. In accordance with our findings, their sensitivity analysis indicated a large within-subject variability of estimated stiffness and an even larger sensitivity of estimated damping.

Apart from the sensitivity issues, one might argue that even though the parameters estimated using a KBI-model may not have a simple relationship with the parameters of the musculoskeletal system, they do reflect ‘some’ of its dynamics. And, if the protocol for stiffness and damping estimation is left unchanged over experimental conditions, one might investigate the (bio-)mechanical variables controlled by the CNS. However, such a point of view raises fundamental problems. For example, as can be appreciated from the Results (see Figure 4), estimated stiffness can change substantially by only changing CE *damping*. CE damping however changes not only with muscle activation, but also with contraction velocity. Therefore, changes in estimated stiffness do not necessarily reflect either changes in CE/SE stiffness due to changed motor commands per se. In addition, CE stiffness is not only influenced by activation level, but also by CE length (and hence tendon length) and activation/contraction history (see e.g. [22]) and therefore also influence the stiffness and damping identified. In conclusion, we are of the opinion that by investigating changes in estimated stiffness and damping using a stiffness-damper-inertia model, even if the experimental protocol is left unchanged, does not provide information about the dynamical properties that are under the control of the CNS.

There is ample evidence that the dynamics of the musculoskeletal system positively contribute to stabilize posture and movement control and that the dynamics are influenced by changes in motor commands (e.g. [4], [6], [9], [10], [26]). However, using a KBI-approach to measure the specific changes in musculoskeletal dynamics that are under the control of the CNS is both theoretically and experimentally problematic. As mentioned in the Introduction, these problems arise because a KBI-model neglects fundamental dynamical properties of the musculoskeletal system. When it comes to analyzing musculoskeletal dynamics, two alternatives are available that might avoid these issues. A first alternative is to use parametric models of higher order than the KBI-model. A third-order model as depicted in this study seems to be of the minimal complexity to capture the dynamics of a muscle and its interaction with the skeleton. Nevertheless, since this model is still less complex than the real system it remains to be investigated whether such a model is free from problems similar to those encountered when using a KBI-model. The difficulty in evaluating the adequacy of a third (or higher) order model is that one needs to compare it to a more complex/realistic model of the (neuro-)musculoskeletal system ([27]–[30]). A second and perhaps better alternative is to make use of nonparametric analysis techniques. The major advantage of such an approach is that it does not require an a-priori assumption about the underlying order of the musculoskeletal system [31], [32]. Yet the disadvantage of such techniques, and the one described before, is that it assumes linearity of the musculoskeletal system [33] and it remains to be shown how well it is capable describing the non-linear dynamics of the real (neuro-)musculoskeletal system.

## Materials and Methods

### Model definition

The dynamics of the musculoskeletal model (MSM, Fig. 1a) and the KBI-model (Fig. 1b) are formulated in terms of their impedance, Z*_MSM_* and Z*_KBI_* respectively, in the Laplace domain (see Supporting Information S1):
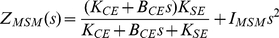
(1)




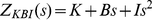
(2)


For convenience, we take the parameters of each of the models in a parameter vector and define ***p_MSM_*** = [*K_CE_ B_CE_ K_SE_ I_MSM_*]^T^ for the MSM, and ***p_KBI_*** = [*K B I*]^T^ for the KBI-model.

### Parameters of the MSM

In essence, the MSM presented in this study acts like a lumped muscle representing all muscles crossing the elbow joint that work basically in series. Parameters of muscles crossing the joint were obtained from the literature [34]–[36] (see also [30], [37], [38]). The range of *K_SE_* estimated was based on a non-linear quadratic spring and its stiffness can be written as:
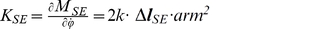
(4)


With *k* the tendon stiffness, Δ*l_SE_* the tendon elongation, *M_SE_* the torque due to tendon force and *arm* the moment arm of the muscle. *k* was calculated such that at maximal isometric CE force, SE elongates 4%. At zero CE force (Δ*l_SE_* = 0), *K_SE_* is zero. Using the parameters of the individual muscles crossing the elbow joint, total *K_SE_* at maximal isometric CE force was estimated to be 1800 Nm/rad.

The CE force-length relationship mainly originates from the changes in myofilament overlap and (at maximal muscle activation) is reasonably well described by a parabola. In this case *K_CE_* is linearly dependent on *l_CE_*:
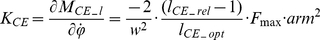
(5)


With *w* a parameter defining the active and passive slack length of a muscle, *l_CE_rel_* the CE length relative to its optimum CE length (*l_CE_opt_*) and *M_CE_l_* the torque due to the CE force-length relationship. Based on the sliding filament theory, this parameter was set to 0.56, yielding a maximal *l_CE_rel_* range of 1±*w*. Maximal *K_CE_* (at active slack) for a lumped elbow muscle is about 200 Nm/rad. Obviously, at optimum CE length no stiffness originates from changes in myofilament overlap (the derivative of force-length relationship = 0) and should be seen as an upper limit. In reality, CE stiffness increases due to short-range stiffness [19] and sarcomere length dependent [Ca^2+^] sensitivity [20]. In addition, experimental studies indicate that the steady state muscle force after slow stretch is higher than expected solely on the basis of the myofilament overlap function [39].

Damping parameters of the joint are not readily obtained from the literature, partially because these parameters are often based on the KBI approach and partially because damping of individual muscle is not often reported. We based *B_CE_* on considerations of the classic Hill-type (concentric) force-velocity relationship:
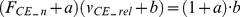
(6)


*F_CE_n_* is the normalized CE force; a and b are the Hill parameters and were set to 0.41 and 5.2 respectively (b/a is the maximal contraction velocity); *v_ce_rel_* is the normalized contraction velocity (normalized to *l_CE_opt_*). Taking the derivative of *F_CE_n_* with respect to *v_CE_rel_*, rewritten in terms of joint damping and scaled with the maximal CE force, *B_CE_* is expressed as:
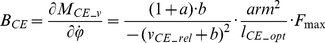
(7)


Estimated maximal concentric (lumped muscles) elbow joint damping (*B_CE_*) on the basis of a Hill-type muscle was about 18 Nms/rad. The eccentric force-velocity slope at *v_CE_* = 0 is estimated to be about twice as high as that of the concentric part [40], yielding a *B_CE_* of 36 Nms/rad.

For graphical purposes and based on the rationale that *K_SE_* cannot be set independently from muscle CE force, *K_SE_* was chosen to be a fixed value of *K_CE_*. Around active slack, *K_CE_* is maximal, but because *F_CE_* is zero *K_SE_* is very low. On the other hand, at *l_CE_opt_*, no stiffness originates from changes in myofilament overlap (derivative of force-length relationship = 0) and hence SE stiffness is much greater that CE stiffness. At a relative CE length of .8 times optimum length, CE stiffness of a lumped elbow muscle maximal stimulation is about 80 Nm/rad. This value increases in reality due to LDCS and short range stiffness and hence maximal CE stiffness was set to an upper limit of 100 Nm/rad (it can be even higher at shorter CE lengths). At this CE length and at maximal activation, SE stiffness is about 10 times higher. At the same CE length but with an activation yielding a *K_CE_* of about 30 Nm/rad, *K_SE_* is about 4 times that of the CE. At a CE stiffness of 10 Nm/rad, *K_SE_* equals that of the CE. This all taken into account, we used the following parameter set:

- *I_MSM_* = 0.10 kgm^2^ (the inertia of the forearm relative to the elbow joint)
- *K_CE_* = 10−200 Nm/rad
- *K_SE_* = 2, 5 and 10×*K_CE_*
- *B_CE_* = 2−32 Nms/rad

### Numerical optimisation of the response match

Simulations were carried out to obtain the responses to perturbations (impulse, sinusoid, pulse, double pulse) of the MSM (*ϕ_MSM_* ) using values of ***p_MSM_***. For each of the combinations of ***p_MSM_***, optimal values for ***p_KBI_*** ^*^ = [*K* ^*^ *B* ^*^ *I* ^*^ ] were identified that minimized the difference between *ϕ_MSM_* and response of the KBI-model (*ϕ_KBI_*). Optimal values were identified for three different time intervals over which the responses were fitted (T = 50, 100 or 200 ms). These intervals were based on the range reported in the literature (e.g. 40–140 ms [10]; 50 ms [12], [41]; 60 ms [15]; 150 ms [14], [42]. ***p_KBI_*** was optimized by minimizing the following error criterion that is only sensitive to differences in the shape of the responses
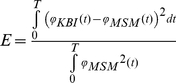
(3)


The value of *E* obtained with ***p_KBI_*** ^*^ will be indicated as E^*^ (see Figure 6) and corresponds to the (minimal) value of the error criterion function for a given set of ***p_MSM_***.

### Analytical approximations

To gain insight in the relation between parameters of the MSM and those of the optimized KBI-model, analytical approximations of the impedance of the MSM were derived for low-frequency (Z*_LF_*; s = 0) and for high-frequency (Z*_HF_*; s = ∞) perturbations. The low-frequency approximation that results from Taylor expansion of the impedance of the MSM (*s* = 0) is:
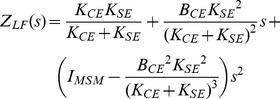
(8)


In line with the numerical approximation of *Z_LF_*, *Z_HF_* can be obtained by the Taylor expansion of the impedance of the reference model for *s* = ∞:
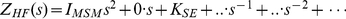
(9)


Not surprisingly, at high perturbation frequencies inertia dominates the dynamic behavior. At *s* = ∞, any CE damping resists the CE from length changes and hence yields zero overall damping. In more practical situations, perturbation frequencies are never so high that the damping of the musculoskeletal system is completely removed. A better approximation of the impedance (when *s* is high, but not infinite) was based on the rationale that at high frequency perturbations, CE force is dominated by CE damping and as such CE stiffness was removed from the impedance of the MSM:
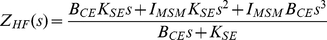
(10)


Second, as high frequency conditions are characterized by large magnitudes of the Laplace variable s, the term *B_CE_s* will be large with respect to *K_SE_*. Ignoring *K_SE_* from Eq. 10 yields:
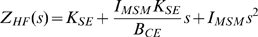
(11)


This approximation is identical to the formal expansion for high frequency perturbations with an additional damping term and it was found that this approximation markedly improved the description of the impedance at high frequency conditions. Below, these analytical expressions will be used to analyze the relationship between the parameters of the MSM and KBI-model under low and high frequency conditions.

### Sensitivity analysis

In an experiment, inherent errors occur in both the applied perturbation and the measured response. To directly explore the sensitivity of estimated stiffness and damping to such errors, a sensitivity analysis was carried out. Any given difference between actual and measured joint angle time histories can be expressed by a corresponding value of the error criterion *E*. In this sensitivity analysis, we calculated the maximal change in parameter values of the KBI-model that led to an arbitrarily small Δ*E*, and hence assessed the sensitivity of parameters that can be expected on the basis of measurement errors.

 >For a given optimally estimated ***p_KBI_*** ^*^ at the minimum of the error function (E^*^), the first derivative matrix (or Jacobian) of *E* to the optimal parameter set ***p_KBI_*** ^*^ is zero by definition. Therefore, the second derivative (or Hessian, *H* = ∇^2^ *E*) of *E* with respect to ***p_KBI_*** determines the change of *E* as a function of small changes in ***p_KBI_***. Defining small changes from ***p_KBI_***, Δ***p_KBI_*** = [Δ*K* Δ*B* ΔI], Δ*E* can be approximated by a second-order Taylor expansion of *E*: 
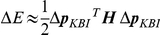



Using this Hessian, the Δ***p_KBI_*** is calculated for which *E* changes the least. Thus, in other words, this Δ***p_KBI_*** indicates the combination of parameter values that can be changed the most before the difference between the response of the MSM and KBI reaches an error Δ*E* (see Figure 6). Since *H* is a symmetrical matrix, the direction of Δ***p_KBI_*** is defined by the eigenvector (***v*** = [*v_1_ v_2_ v_3_*]^T^) corresponding to the *smallest* eigenvalue (λ) of the *H*:
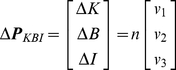
(12)


*n* indicates the norm of Δ***p_KBI_***. Because ***v*** is an eigenvector of ***H*** we have ***Hv*** = λ***v***, and therefore:
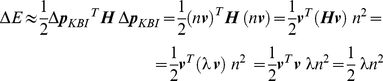
(13)


So the error increase Δ*E* is proportional to the smallest eigenvalue of ***H*** and to the squared norm of Δ***p_KBI_***. Using Eq. 11, Δ*E* can be expressed in terms of Δ***p_KBI_***:
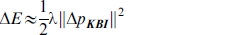
(14)


The largest simultaneous change in parameters as a function of Δ*E* is given by:
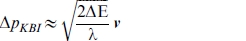
(15)


The sensitivity of stiffness and damping to a difference in measured/optimized response can be readily obtained using Equation 15. Note that ***v*** depends on all parameters of the MSM and thus was calculated for every ***p_MSM_***.

### Computational aspects

All calculations were performed in Matlab (R14). A Nelder–Mead simplex search method [43] was used to identify the values of ***p_KBI_*** ^*^. The Hessians for every ***p_MSM_*** was estimated by computing a finite-difference approximation.

## Supporting Information

Supporting Information S1 (DOCX)

## References

[1] Capaday C, Stein RB (1986). Amplitude modulation of the soleus H-reflex in the human during walking and standing.. J Neurosci.

[2] Prochazka A (1981). Muscle spindle function during normal movement.. Int Rev Physiol.

[3] Houk JC (1979). Regulation of stiffness by skeletomotor reflexes.. Annu Rev Physiol.

[4] De Serres SJ, Milner TE (1991). Wrist muscle activation patterns and stiffness associated with stable and unstable mechanical loads.. Exp Brain Res.

[5] Milner TE (2002). Adaptation to destabilizing dynamics by means of muscle cocontraction.. Exp Brain Res.

[6] Gribble PL (2003). Role of cocontraction in arm movement accuracy.. J Neurophysiol.

[7] Gottlieb GL, Agarwal GC (1978). Dependence of human ankle compliance on joint angle.. J Biomech.

[8] Zinder SM (2007). Validity and reliability of a new in vivo ankle stiffness measurement device.. J Biomech.

[9] Granata KP (2004). Active stiffness of the ankle in response to inertial and elastic loads.. J Electromyogr Kinesiol.

[10] Lacquaniti F, Licata F, Soechting JF (1982). The mechanical behavior of the human forearm in response to transient perturbations.. Biol Cybern.

[11] Bennett DJ (1992). Time-varying stiffness of human elbow joint during cyclic voluntary movement.. Exp Brain Res.

[12] Popescu F, Hidler JM, Rymer WZ (2003). Elbow impedance during goal-directed movements.. Exp Brain Res.

[13] Lacquaniti F, Carrozzo M, Borghese NA (1993). Time-varying mechanical behavior of multijointed arm in man.. J Neurophysiol.

[14] Gomi H, Kawato M (1997). Human arm stiffness and equilibrium-point trajectory during multi-joint movement.. Biol Cybern.

[15] Burdet E (2000). A method for measuring endpoint stiffness during multi-joint arm movements.. J Biomech.

[16] Agarwal GC, Gottlieb GL (1977). Oscillation of the human ankle joint in response to applied sinusoidal torque on the foot.. J Physiol.

[17] Hunter IW, Kearney RL (1982). Dynamics of human ankle stiffness: variation with mean ankle torque.. J Biomech.

[18] Winters JM, Stark L (1988). Estimated mechanical properties of synergistic muscles involved in movements of a variety of human joints.. J Biomech.

[19] Rack PM, Westbury DR (1974). The short range stiffness of active mammalian muscle and its effect on mechanical properties.. J Physiol.

[20] Kistemaker DA, Van Soest AK, Bobbert MF (2005). Length-dependent [Ca2+] sensitivity adds stiffness to muscle.. J Biomech.

[21] Rack PM, Westbury DR (1969). The effects of length and stimulus rate on tension in the isometric cat soleus muscle.. J Physiol.

[22] Herzog W, Leonard TR (2000). The history dependence of force production in mammalian skeletal muscle following stretch-shortening and shortening-stretch cycles.. J Biomech.

[23] Nichols TR, Houk JC (1976). Improvement in linearity and regulation of stiffness that results from actions of stretch reflex.. J Neurophysiol.

[24] Hoffer JA, Andreassen S (1981). Regulation of soleus muscle stiffness in premammillary cats: intrinsic and reflex components.. J Neurophysiol.

[25] Tsujii T, Attardi B, Winters SJ (1995). Regulation of alpha-subunit mRNA transcripts by pituitary adenylate cyclase-activating polypeptide (PACAP) in pituitary cell cultures and alpha T3-1 cells.. Mol Cell Endocrinol.

[26] Gomi H, Osu R (1998). Task-dependent viscoelasticity of human multijoint arm and its spatial characteristics for interaction with environments.. J Neurosci.

[27] Pandy MG (2001). Computer modeling and simulation of human movement.. Annu Rev Biomed Eng.

[28] van Soest AJ, Bobbert MF (1993). The contribution of muscle properties in the control of explosive movements.. Biol Cybern.

[29] Zajac FE (2002). Understanding muscle coordination of the human leg with dynamical simulations.. J Biomech.

[30] Kistemaker DA, Van Soest AK, Bobbert MF (2007). Equilibrium point control cannot be refuted by experimental reconstruction of equilibrium point trajectories.. J Neurophysiol.

[31] Kearney RE, Hunter IW (1990). System identification of human joint dynamics.. Crit Rev Biomed Eng.

[32] Perreault EJ, Crago PE, Kirsch RF (2000). Estimation of intrinsic and reflex contributions to muscle dynamics: a modeling study.. IEEE Trans Biomed Eng.

[33] Perreault EJ, Kirsch RF, Acosta AM (1999). Multiple-input, multiple-output system identification for characterization of limb stiffness dynamics.. Biol Cybern.

[34] Murray WM, Buchanan TS, Delp SL (2000). The isometric functional capacity of muscles that cross the elbow.. J Biomech.

[35] Nijhof E, Kouwenhoven E (2000). Simulation of multijoint arm movements, in Biomechanics and Neural Control of Posture and Movement, J. Winters and P. Grago, Editors..

[36] Murray WM, Delp SL, Buchanan TS (1995). Variation of muscle moment arms with elbow and forearm position.. J Biomech.

[37] Kistemaker DA, Van Soest AJ, Bobbert MF (2007). A model of open-loop control of equilibrium position and stiffness of the human elbow joint.. Biol Cybern.

[38] Kistemaker DA, Van Soest AJ, Bobbert MF (2006). Is equilibrium point control feasible for fast goal-directed single-joint movements?. J Neurophysiol.

[39] Rassier DE, Herzog W (2005). Relationship between force and stiffness in muscle fibers after stretch.. J Appl Physiol.

[40] Katz B (1939). The relation between force and speed in muscular contraction.. J Physiol.

[41] Wong J (2009). The Influence of Visual Perturbations on the Neural Control of Limb Stiffness.. Journal of Neurophysiology.

[42] Simoneau M, Denninger M, Hain TC (2008). Role of loading on head stability and effective neck stiffness and viscosity.. J Biomech.

[43] Lagarias JC (1998). Convergence properties of the Nelder-Mead simplex method in low dimensions.. Siam Journal on Optimization.

