# Supplementary material for: In Vivo Dynamics of the Musculoskeletal System Cannot Be Adequately Described Using a Stiffness-Damping-Inertia Model
Source: PLoS One. 2011 May 27;6(5):e19568. doi: 10.1371/journal.pone.0019568 (PMC3103502; doi:10.1371/journal.pone.0019568)
Supplement: Supporting Information S1 — (DOCX) [file pone.0019568.s001.docx]

**Supporting Information I**

*Results*


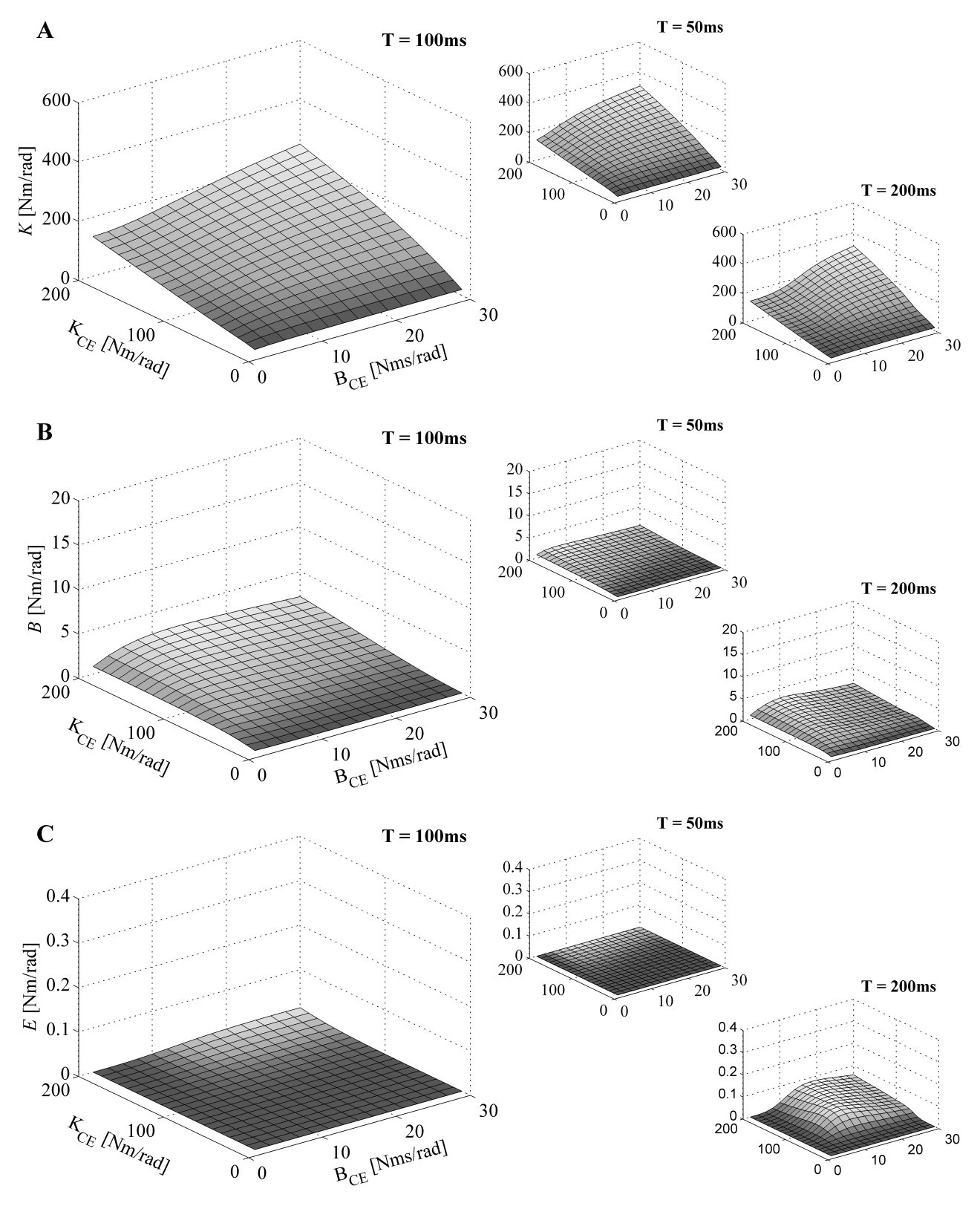


Figure S3 Estimated stiffness *K* (A), damping *B* (B) and matching error *E* (C) for KSE =2 × *K_CE_* for three time windows (50, 100, and 200 ms). Impulse torque perturbation.


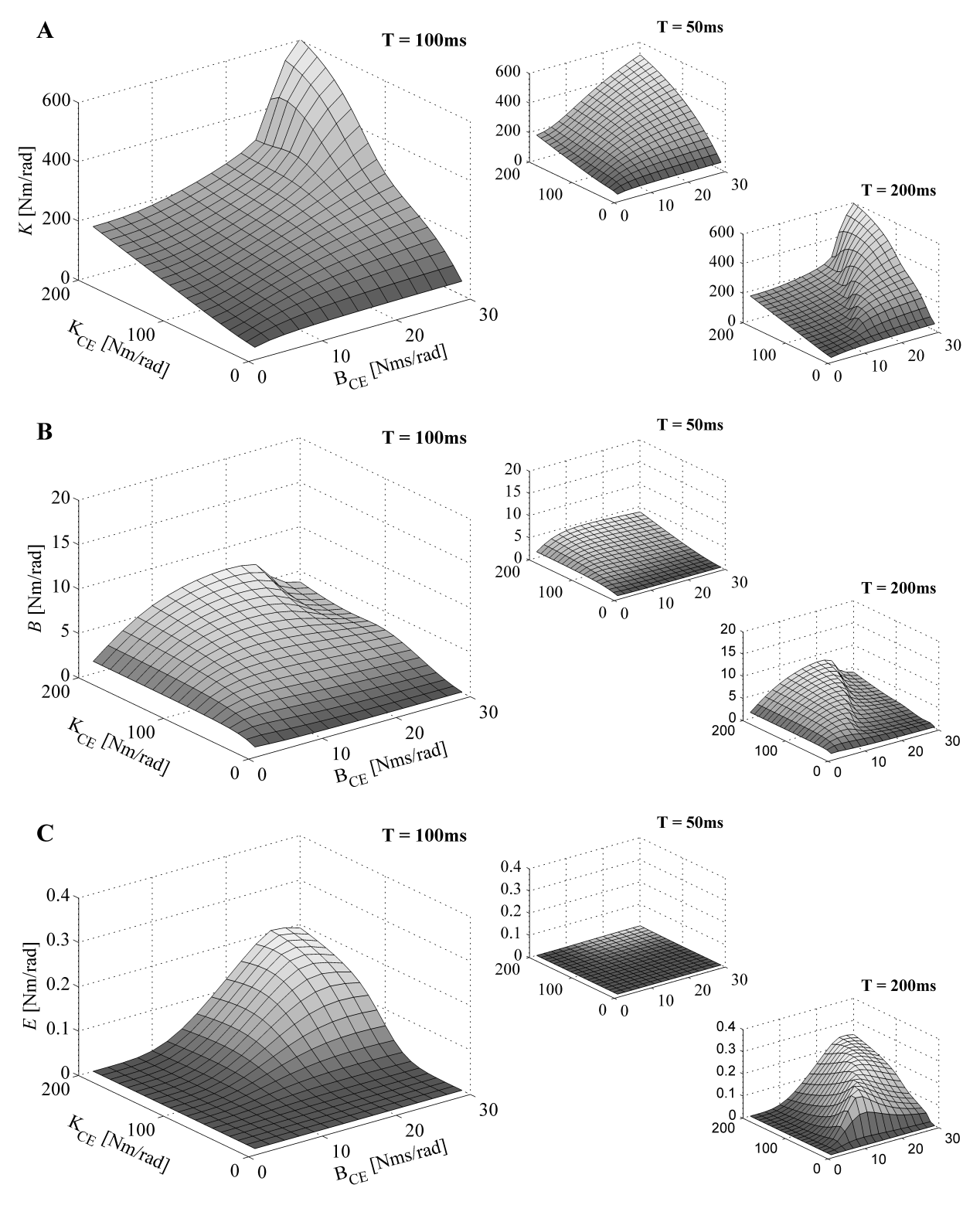


Figure S4 Estimated stiffness *K* (A), damping *B* (B) and matching error *E* (C) for KSE =5 × *K_CE_* for three time windows (50, 100, and 200 ms). Impulse torque perturbation.


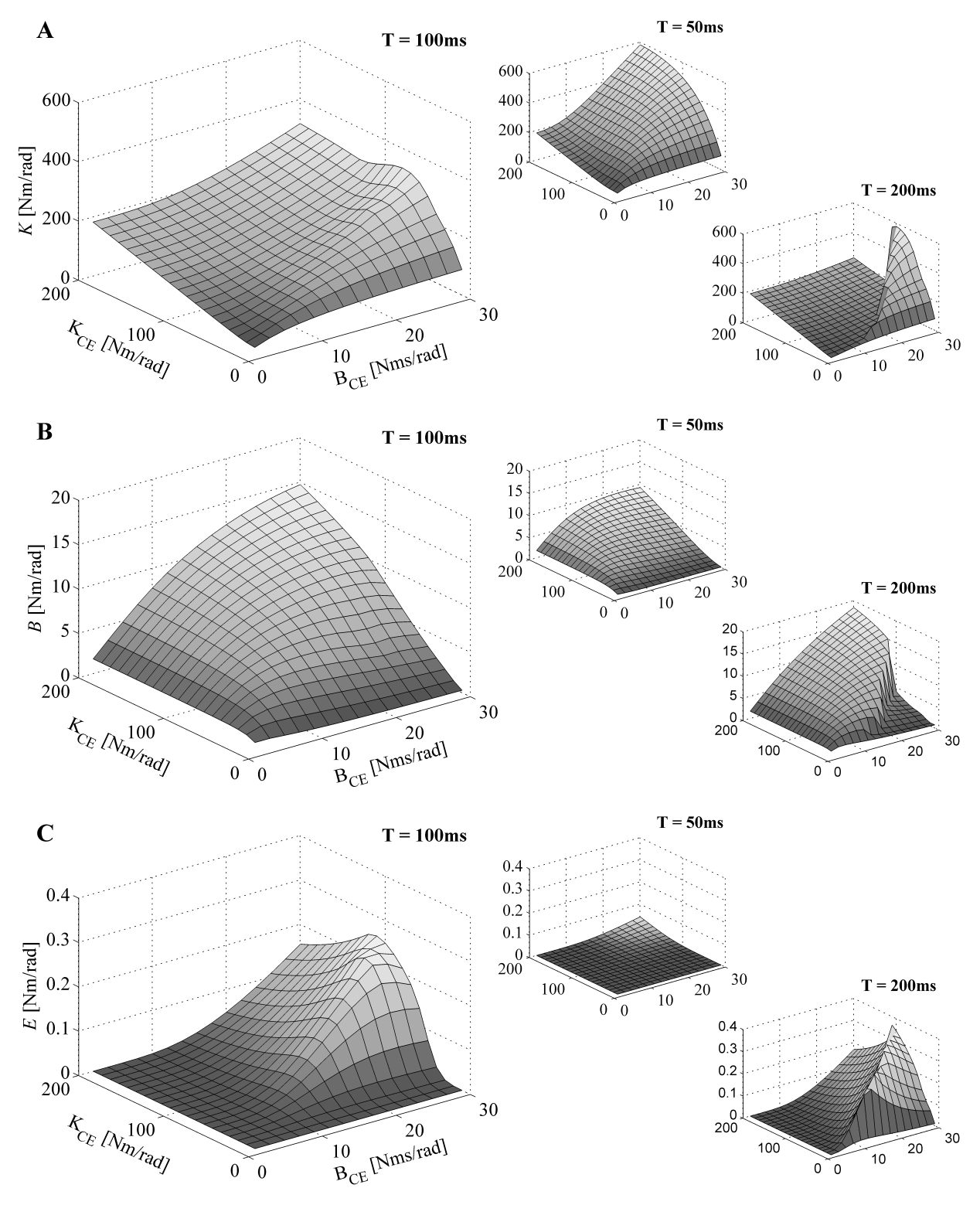


Figure S5 Estimated stiffness *K* (A), damping *B* (B) and matching error *E* (C) for KSE =10 × *K_CE_* for three time windows (50, 100, and 200 ms). Impulse torque perturbation.

*Methods*


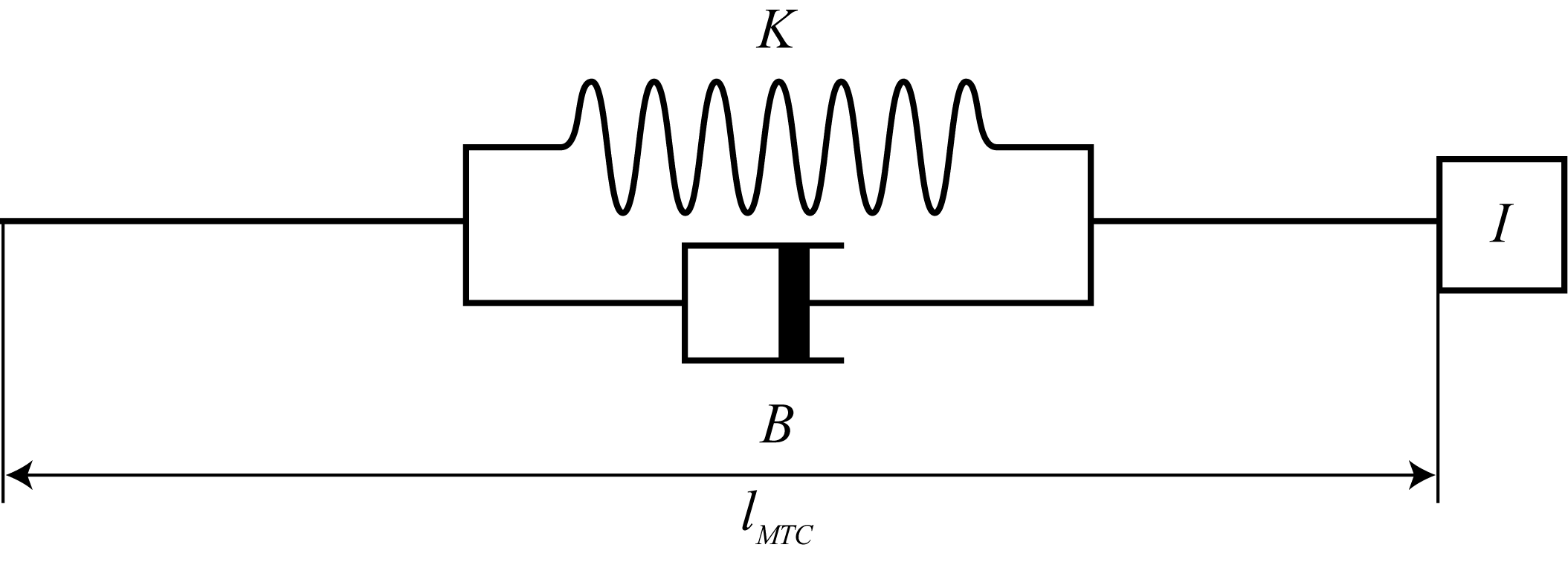


Figure S1. Graphical representation of the KBI-model.

The dynamics of the KBI-model (Figure S1) are obtained by Newton’s dynamic equation of motion for a rotational body. Using that the moment arm times *l_MTC_* equals the joint angle (*φ*), we obtain for the KBI-model:

 (S1)

with the angle, *K* the stiffness, *B* the damping, *I* the rotational inertia and *M_ext_* the external (perturbation) torque. Transforming equation S1 to the Laplace domain:

$$\Sigma T=I\Phi s^{2}=-K\Phi-B\Phi s+T_{ext}$$

with *T*(*s*) the Laplace transformed torque and Φ(*s*) the Laplace transformed angular displacement. The impedance in the Laplace domain is given by:

$Z_{KBI}(s)=\frac{T_{ext}(s)}{\Phi(s)}=K+Bs+Is^{2}$ (S2)

with *s* the Laplace operator.


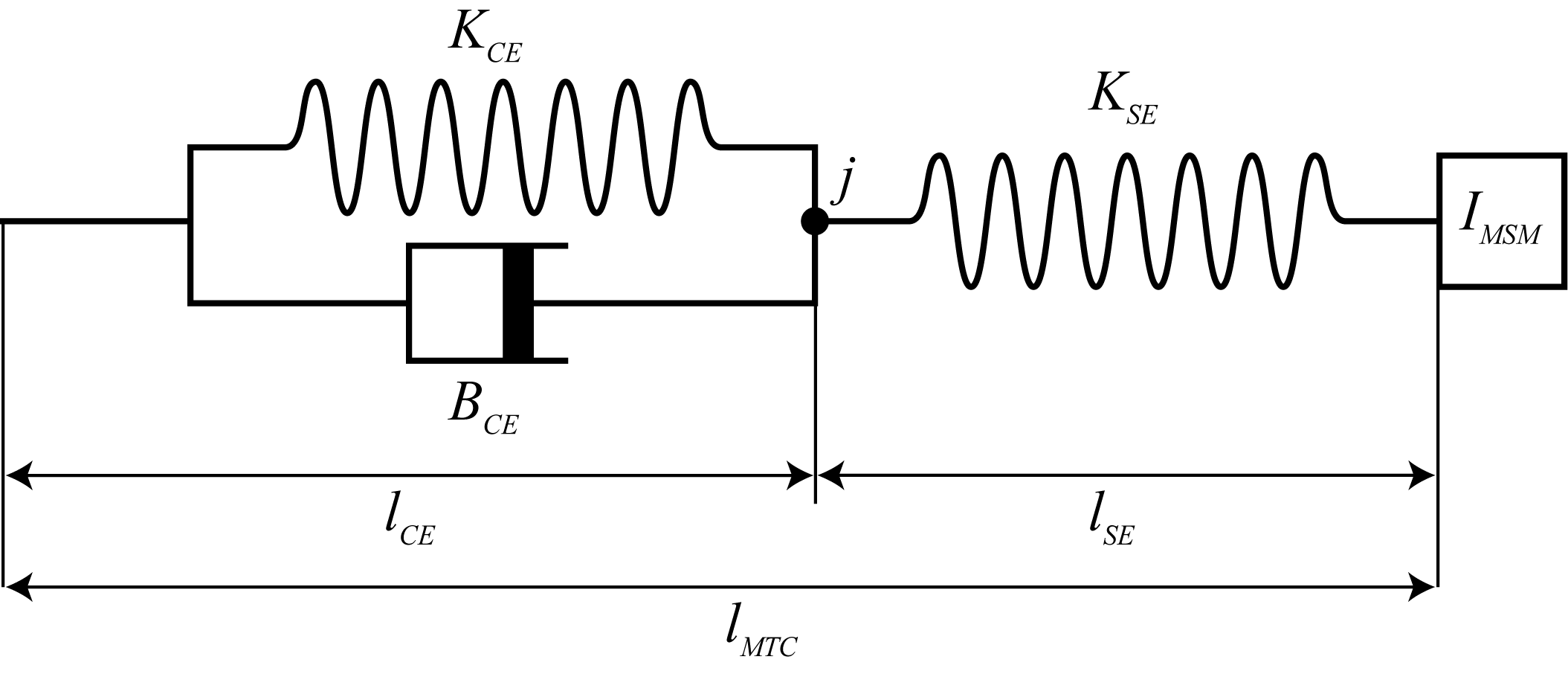


Figure S2. Graphical representation of the linearized Hill-type MSM.

The dynamic equations for the MSM are less readily obtained as contraction dynamics within the muscle arise from the force-velocity relationship (damper) of the CE that works in series with a tendon (spring). By assuming that the inertia of the muscle itself is negligible with respect to the forces produced by the muscle, we obtain for the contraction dynamics:

 (S3)

with *F_j_* the forces in the CE-SE junction (*j*), *l_CE_* and *l_SE_* length of CE and SE, respectively, *K_CE_* and *B_CE_*_­_ stiffness and damping of CE, and *K_SE_* stiffness of SE. When transformed to the Laplace domain, we get:

$-K_{CE}L_{CE}-B_{CE}L_{CE}s+K_{SE}L_{SE}=0$ (S4)

with *L_CE_* and the *L_SE_* the Laplace transformed *l_CE_* and *l_SE_*. Using that the total length of the muscle-tendon complex (*l_MTC_*) equals *l_CE_* + *l_SE_* (see Figure S2), *L_SE_*(*s*) can be expressed as a function of *L_MTC_*(*s*):

$L_{SE}=\frac{(K_{CE}+B_{CE}s)}{K_{CE}+B_{CE}s+K_{SE}}L_{MTC}$ (S5)

For the equations of skeletal motion we obtain:

 (S6)

with *I_MSM_* the rotational inertia of the MSM and *arm* the moment arm of the muscle. When transformed to the Laplace domain Equation S6 reads:

$\Sigma T=I_{MSM}\Phi s^{2}=-K_{SE}L_{SE}arm+T_{ext}$ (S7)

By substituting *L_SE_* in Equation S5 in S7 and using that *l_MTC_*×Δarm = Δ*φ*, the impedance of the MSM (*Z_MSM_*(*s*)) is given by:

$Z_{MSM}(s) =\frac{(K_{CE}+B_{CE}s)K_{SE}}{K_{CE}+B_{CE}s+K_{SE}}+I_{MSM}s^{2}$ (S8)
